# Regulation of TGF-**β**2-induced epithelial–mesenchymal transition and autophagy in lens epithelial cells by the miR-492/*NPM1* axis

**DOI:** 10.17305/bb.2024.10249

**Published:** 2024-10-01

**Authors:** Yanqiong Bao, Guangjie Ding, Haiqing Yu, Yawei He, Jiayan Wu

**Affiliations:** 1 Department of Ophthalmology, Zhenhai Longsai Hospital, Zhejiang, China; 2 Department of Health Management Center, Zhenhai Longsai Hospital, Zhejiang, China

**Keywords:** Cataract, Nucleophosmin 1 (NPM1), transforming growth factor β2 (TGF-β2), epithelial–mesenchymal transition (EMT), miR-429, autophagy.

## Abstract

A cataract is a clinically common blinding disease closely related to the aging of the eye cells, which has become a major health killer in the elderly. Our research seeks to analyze the primary targets linked to the pathogenesis of cataracts during the aging process. We performed bioinformatics analyses on the GSE101727 dataset to discover genes linked with aging and cataracts. To explore the impacts of Nucleophosmin 1 (*NPM1)* on cell apoptosis, proliferation, as well as epithelial–mesenchymal transition (EMT) processes, in vitro tests, such as western blotting, flow cytometry, and MTT, were carried out. Additionally, the study incorporated transforming growth factor **β**2 (TGF-**β**2) to examine its function in cellular responses, chloroquine (CQ) to regulate autophagic flow, and H_2_O_2_ therapy to mimic oxidative stress. Our study discovered seven aging-related genes, including *NPM1*, that had substantial relationships with cataracts. *NPM1* overexpression was shown to boost cell proliferation and prevent apoptosis in SRA01/04 cells. Notably, *NPM1* modulated the TGF-**β** signaling pathway, influencing cell proliferation and EMT processes. miR-429 was shown to be adversely regulating *NPM1* and autophagy-related proteins, as demonstrated by changes in their expression in response to TGF-**β**2 treatment. Furthermore, *NPM1* knockdown restored autophagy activity suppressed by miR-429 mimics, indicating a complex interaction of miR-429, *NPM1*, and TGF-**β**2 pathways in regulating autophagy and EMT. Lens epithelial cell proliferation and apoptosis were largely regulated by *NPM1*, as well as autophagy and EMT, which were significantly mediated by TGF-**β**2 and the miR-429/*NPM1* axis. These results implied new possible targets for prognosis and therapy of cataracts.

## Introduction

Cataract is a visual disorder with a gradual onset caused by lens opacity, mainly manifested as painless progressive vision loss [1]. The disease frequently occurs in people over 50 years old, and senile cataracts account for 25%–50% of blinding diseases [2]. According to the clinical manifestations of patients, we divided cataracts into three stages, early, middle, and advanced stages. Among them, the early symptoms are generally not obvious, only mild blurred vision [3]. In the middle term, the lens opacity of the patient gradually increases, and abnormal sensations like myopia and glare may occur [4]. Finally, in the advanced stage, the patient becomes completely blind [5]. Currently, surgery is the only effective way for cataract patients [6, 7]. Li et al. [8] mentioned in the literature that Brg1 is not only related to the formation of cataracts but also regulates the expression of different genes, such as *HSP90AB1* and *POLR2E*, to affect the occurrence and development of cataracts. In addition, Zhang et al. [9] also found four genes related to Crim1-induced congenital cataract molecular pathogenesis, namely, *C1qa*, *C1qb*, *C1qc*, and *Cd74*. In this paper, we aimed to explore new clinical biomarkers and therapeutic methods for cataract patients.

Nucleophosmin 1 (NPM1) is a multifunctional protein that is overexpressed in actively proliferating cells and cancer cells, where it exerts its oncogenic effects by binding and inhibiting various tumor suppressors [10]. It is mainly localized in the nucleolus, but it also shuttles between the nucleus and cytoplasm, exhibiting diverse cellular functions [11]. It plays a crucial role in the maintenance of normal cellular functions, and alterations in its regulation, whether through overexpression, mutation, translocation, loss of function, or sporadic deletion, may lead to cancer and tumorigenesis. Such effects may involve key biological processes such as ribosome biogenesis, chromatin remodeling, genome stability, cell cycle progression, and apoptosis [12]. Studies have demonstrated that acute myeloid leukemia (AML) and *NPM1* mutations are related [13, 14]. For example, a study by Falini et al. demonstrated that adult AML is most frequently caused by *NPM1* mutations, which induce abnormal cytoplasmic delocalization in *NPM1* mutants [15]. Luchinat et al. elaborated that P14ARF activity is regulated by NPM1, which controls both its levels and cellular localization. In AML with *NPM1* mutations, mutant NPM1 is abnormally translocated in cytoplasmic lysates carrying P14ARF and subsequently degraded, thereby impairing the P14ARF-HDM2-p53 axis [16]. Hindley et al. [17] revealed the structure and normal cellular function of *NPM1*, its common mutation types, as well as the mechanisms by which it affects AML development and progression. However, there is no research related to *NPM1* and cataracts yet.

One essential cellular signaling cascade that controls a variety of biological activities is the transforming growth factor-β (TGF-β) signaling pathway [18]. Within the multifunctional growth factor family, including members like TGF-β1, TGF-β2, and TGF-β3 [19], the second one stands out as a critical participant known for its involvement in epithelial-to-mesenchymal transition (EMT) and its effects on cellular proliferation, differentiation, and apoptosis [20]. A study has found that in the context of neovascular age-related macular degeneration (AMD), TGF-β2 plays a prominent role by inducing pericyte–myofibroblast transition (PMT) via the Smad2/3 and Akt/mTOR pathways [21]. This underscores the crucial involvement of PMT in subretinal fibrosis, emphasizing its potential as a therapeutic target. Furthermore, a new study has revealed a close association between the aberrant TGF-β2 expression and signaling and the pathogenesis of high intraocular pressure and primary open-angle glaucoma. Particularly, TGF-β2 activates both classical (Smad) and non-canonical (MAPK, Rho GTPase) signaling pathways in trabecular meshwork cells [22]. Additionally, another investigation has unveiled that TGF-β2 induces the overexpression of circ-PRDM5 in lens epithelial cells, promoting EMT via the circ-PRDM5/miR-92b-3p/*COL1A2* axis [23]. Previous studies in this field have shown that a TGF-β2 concentration of 10 ng/mL is suitable for producing the desired biological effects. This concentration has been used extensively in similar studies and has been reported in the literature. It was found that E-calmodulin was significantly downregulated, whereas waveform proteins were upregulated, after treatment of cells with 10 ng/mL TGF-β2 for 24 h, and this treatment induced EMT in human lens epithelial-B3 cells [24]. In addition, we found that EMT was promoted in primary mouse retinal pigment epithelium (RPE) cells under stimulation with the same TGF-β2 concentration of 10 ng/mL, thereby promoting subretinal fibrosis [25]. Interestingly, when we performed chemical induction of ER stress under conditions of TGF-β2 stimulation, we observed that this process inhibited EMT and migration in RPE cells, suggesting that it may be through inactivation of TGF-β signaling [26]. Therefore, a thorough understanding of TGF-β function in the signaling pathway is essential for the advancement of cataract research.

MicroRNAs (miRNAs) are a class of non-coding RNAs consisting of 18–24 nucleotides that transcriptionally downregulate gene expression and regulate the expression of 20% or more of the human genome [27]. The miRNA transcriptomes of the mammalian retina [28], lens [29], and cornea [30] have been identified and characterized. Despite these advances, however, much remains unknown about the function and pathophysiologic role of miRNAs in ophthalmology [31]. miR-429 is a member of the miR-200 family that is dysregulated in different types of cancer. It has been shown to play a role in inhibiting EMT, tumor metastasis, and chemotherapy resistance [32]. miR-429 ameliorates cartilage damage in OA by targeting FEZ2 and promoting autophagy [33]. Recent research has highlighted the involvement of miR-429 in regulating endothelial cell migration and the expression of proteins associated with tight junctions [34]. These insights emphasized the multifaceted role of miR-429 and its therapeutic potential in a variety of pathologies, including ophthalmic pathologies.

The GSE101727 dataset was retrieved for this research, and via extensive bioinformatics analysis, the key aging-related gene in the etiology of cataracts, *NPM1*, was identified. The influence of *NPM1* on lens epithelial cell proliferation as well as apoptosis under oxidative stress was then explored further in cell studies, demonstrating its relationship with TGF-β2 in controlling autophagy and EMT. We also identify the miR-429/*NPM1* axis as a major regulator in this relationship, emphasizing its potential therapeutic significance. The diagnosis, course of therapy, and prognosis of cataract patients will all benefit from these discoveries.

## Materials and methods

### Screening and enrichment analysis of differentially expressed genes (DEGs)

The GSE101727 microarray dataset was submitted to the GEO database by Xie et al. Based on the GEO2R tool in the GEO database (https://www.ncbi.nlm.nih.gov/geo/), we screened DEGs in ten aging-related cataract and ten glaucoma patients (*P* < 0.001). The standard for upregulated DEGs was fold change (FC) > 2, and the standard for down-regulated DEGs was FC < 0.5. We used the “clusterProfiler” package to perform enrichment analyses of Gene Ontology (GO) terms and Kyoto Encyclopedia of Genes and Genomes (KEGG) pathways with a significance threshold of *P* < 0.05 to study the biological functions of the DEGs discovered.

### Enrichment analysis of aging-related genes in GSE101727 dataset

Gene set enrichment analysis (GSEA; https://www.gsea-msigdb.org/gsea/index.jsp) is a computer approach for analyzing gene expression data based on a database of molecular features [35]. We downloaded 313 aging-related genes (MSigDB geneset: MSigDB version 7.0 GOBP_AGING) on the GSEA website. With the help of the “VennDiagram” tool, overlapping genes were identified from 313 aging-related genes and GEE101727 DEGs. Then, we used the Search tool for the retrieval of interacting genes (STRING, https://www.string-db.org/) website (default value [i.e., 0.4] moderate confidence) to construct a protein–protein interaction (PPI) network for the overlapping genes obtained from the analysis, and genes with correlations were identified from it. Next, the cluster distribution of these genes in different samples of the GSE101727 dataset was analyzed. Subsequently, GO and KEGG analyses were conducted on these genes.

### Expression and receiver operating characteristic (ROC) analysis of seven aging-related genes

To further understand the prognostic value of seven genes for cataract patients, we confirmed the relative expression levels of these genes in ten cataract (case group) and ten glaucoma patients (control group) in the GSE101727 dataset. Then, the prognostic predictive ability of these genes was identified by ROC curves by using the R package “time ROC” (R-4.1.2-win) as well as associated area under the curve (AUC) values followed by confidence intervals (CI) calculation. A higher AUC value indicated a greater degree of prediction accuracy.

### Wikipathway and Reactome pathway analysis of *NPM1*

In our study, we adopted the GSEA approach to elucidate the biological pathways related to *NPM1*. Two comprehensive pathway databases were used: WikiPathways and Reactome pathways. For GSEA, gene expression profiles related to *NPM1* expression were analyzed, combining gene sets from Wikipathways and Reactome. *P* values were calculated to determine the statistical significance of enriched pathways.

### Cell culture and transfection

Biovector (Beijing, China) provided human lens epithelial cells (SRA01/04), which were cultured at 37 ^∘^C in DMEM with 10% FBS, 1% penicillin/streptomycin, and 5% CO_2_. Transfection procedures followed the manufacturer’s protocol using Lipofectamine^®^ 2000 to introduce the *NPM1* overexpression vector (pcDNA3.1-*NPM1*) and a corresponding empty vector control. Additionally, we incorporated a set of *NPM1* knockdown sequences, including si-*NPM1*-1, si-*NPM1*-2, and si-*NPM*1-3, to assess the effects of *NPM1* suppression. Further transfection procedures were used to introduce miR-429 mimics and inhibitors into SRA01/04 cells to modify the expression of the miRNA.

### Cell treatment

First, cells were treated with different doses (0–300 µM) of hydrogen peroxide. Additionally, to interrogate the potential autophagic response, cells were treated to varying concentrations of chloroquine (CQ) at 0, 50, and 100 µmol/L for periods of 12, 24, and 48 h, respectively. In a separate set of experiments designed to explore the pathway-specific response, cells were treated with 10 ng/mL TGF-β2 without a designated time frame to assess the implications of TGF-β signaling inhibition. In this context, Galunisertib was utilized to further delineate the function of TGF-β signaling in cellular physiology.

### Transmission electron microscopy (TEM)

We used TEM to observe TGF-β2 affected SRA01/04 cells. After being exposed to 10 ng/mL TGF-β2, the cells were post-fixed with osmium tetroxide, fixed with glutaraldehyde, dehydrated in ethanol, and then embedded in resin. To locate and record autophagic vacuoles and other structural alterations, ultrathin sections were stained and seen under a TEM.

### Quantitative real-time PCR (qRT-PCR) analysis

Cultured cells and tissue specimens underwent total RNA isolation using TRIzol Reagent, followed by cDNA generation employing the BeyoRT II cDNA Synthesis Kit, adhering to the provided protocol by the manufacturer. SYBR-Green PCR Master Mix Kit was used to run qRT-PCR using the ABI 7900 Detection System. For mRNA analysis, GAPDH acted as the internal control. Meanwhile, the miR-429 expression study used U6 as the internal reference. PCR data were analyzed employing the 2^−ΔΔCT^ technique. The primer sequences used for polymerase chain reaction analysis are shown in Table S1.

### Cell counting kit-8 (CCK-8) assay

Ninety six-well dishes with SRA01/04 cells were seeded and grown for the specified periods of time. Each well received 10 µL of CCK-8 solution after transfection, which was followed by a 2-h incubation period at 37 ^∘^C. Ultimately, using a microplate reader to measure the optical density (OD) value at 450 nm at 0, 24, 48, 72, and 96 h, the cell proliferation activity was examined.

### Western blotting (WB) analysis

First, cells were destroyed utilizing a protease inhibitor cocktail-containing RIPA solution. The BCA reagent was then used to measure total protein concentration. Following processing on a 10% separating gel and transport to a polyvinylidene difluoride (PVDF) membrane, a 30 µg protein sample was used. After blocking the membrane with 5% skim milk, the membrane was incubated with primary antibodies against NPM1, E-cadherin, ZO-1, vimentin, α-SMA, Bax, Bcl-2, Caspase-3, Smad1, Smad2, TGF-β2, Snail, Slug, ZEB1, LC3-I, LC3-II, P62, Beclin1, ATG7, and GAPDH at a concentration of 1:1000. The secondary antibody was then conjugated with horseradish peroxidase to detect mouse IgG. Software called Quantity One (Bio-Rad) was used to quantify band levels. Loading control was performed using GAPDH.

### Flow cytometry

After culturing SRA01/04 cells post-transfection in a 24-well plate with triplicates for each condition, the cells were fixed overnight in 75% ethanol upon reaching 90% confluency. Following fixation, cells were harvested and resuspended. Subsequently, they underwent triple staining with 5 mL of propidium iodide (PI) and annexin V-fluorescein isothiocyanate (FITC). Finally, cell apoptosis rates were quantified using a flow cytometer (BD Biosciences, USA).

### Dual-luciferase reporter assay

According to bioinformatics predictions, the *NPM1* 3′UTR predicted binding sequences for miR-249 were amplified and inserted into pmirGLO vectors (Promega, Madison, WI, USA). Using Lipofectamine^®^ 3000, the miR-249 mimic and both *NPM1* wildtype (WT) and mutated (MUT) luciferase reporter vectors were co-transfected. The activity of luciferase was measured 48 h thereafter with a dual-luciferase system (Promega).

**Figure 1.**
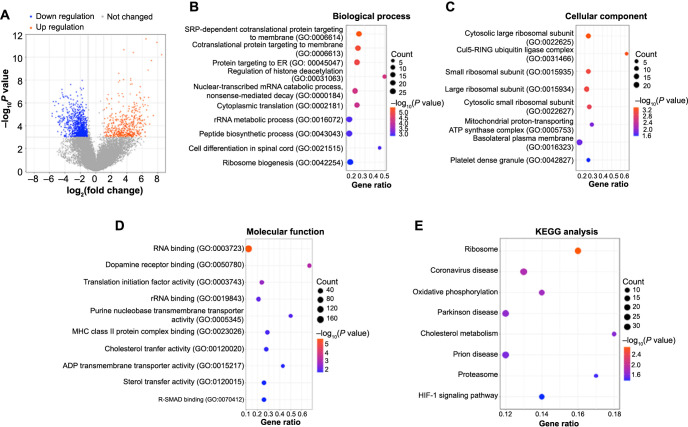
**Screening and enrichment analyses of GSE101727-DEGs.** (A) Volcano plot of genes in the GSE101727 dataset, blue scatters are downregulated DEGs and orange scatters are upregulated DEGs. DEGs enrichment items in (B) BP, (C) CC, (D) MF, and (E) KEGG. The larger the bubble, the greater number of genes, and *P* value represents a significant degree of enrichment. DEGs: Differentially expressed genes; KEGG: Kyoto Encyclopedia of Genes and Genomes.

**Figure 2.**
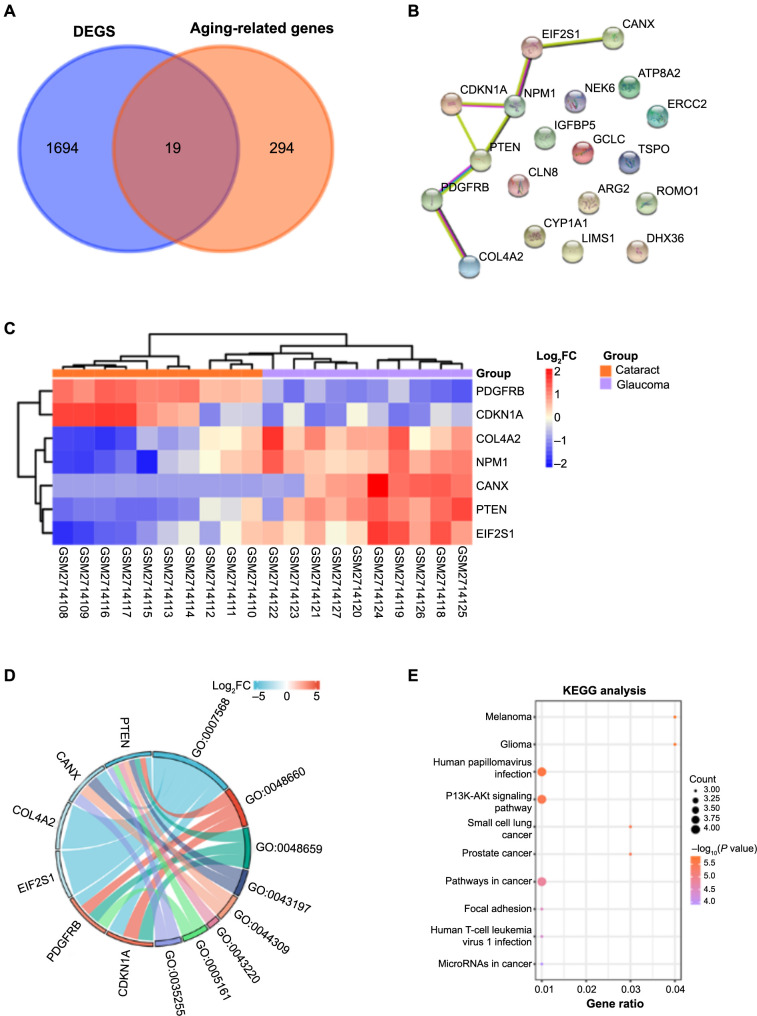
**The overlapping genes between GSE101727-DEGs and aging-related genes.** (A) Venn diagram, 19 genes are overlapped between DEGs and aging-related genes; (B) PPI network analysis; nodes represent genes, and edges represent correlations between genes; (C) Heatmap, clustering of seven genes in GSE101727 dataset samples. The legend represents the *Z*-score; (D) GO analysis, the relevant enrichment items of the six genes in the GO term, the left side is the relative order of the genes according to log_2_FC, and the right side is the order of the enrichment items according to the strong and weak changes; (E) Bubble plot, top ten KEGG pathways for seven gene enrichments. DEGs: Differentially expressed genes; PPI: Protein–protein interaction; GO: Gene ontology; KEGG: Kyoto Encyclopedia of Genes and Genomes.

### MTT assay

MTT tests were used to determine the cell viability. In a 96-well plate, cells were planted at an optimal density of 5000 cells/well. After a 24-h interval, the cells were treated with diluted hydrogen peroxide at the specified concentration. Following the incubation periods, each well received 20 µL of a 5 mg/mL MTT solution. Each well received 150 µL of dimethyl sulfoxide after a 4-h incubation period. Using a Tecan fluorescence microplate reader, the absorbance was determined at 490 nm following 15 min of shaking.

### Statistical analysis

The data was statistically analyzed using Graph Pad Prism 5 software, and the results were reported using means and standard deviations. The Student’s *t*-test was used to investigate differences between two groups, and one-way variance was used to assess differences between several groups. *P* < 0.05 was used to determine the statistical significance of the research.

## Results

### Acquisition and functional enrichment analysis of DEGs related to cataract

In the GSE101727 dataset, according to the set screening criteria, we obtained 486 upregulated (orange scatters) DEGs and 1227 downregulated (blue scatters) DEGs (Figure 1A). The “clusterProfiler” package of the R language performs a series of functional enrichment analyses on DEGs. In the BP category, the DEGs exhibit enrichments in terms such as protein targeting to ER, rRNA metabolic process, and peptide biosynthetic process, as illustrated in Figure 1B. Within the CC classification, DEGs displayed significant enrichments in the large ribosomal subunit, cytosolic small ribosomal subunit, and platelet dense granule, as depicted in Figure 1C. In the MF, DEGs showed enrichments in terms such as dopamine receptor binding, rRNA binding, and translation initiation factor activity, as illustrated in Figure 1D. In addition, in the KEGG pathway, the top eight pathways enriched by DEGs were ribosome, coronavirus disease, oxidative phosphorylation, Parkinson’s disease, cholesterol metabolism, prion disease, proteasome, and HIF-1 signaling pathway (Figure 1E).

**Figure 3.**
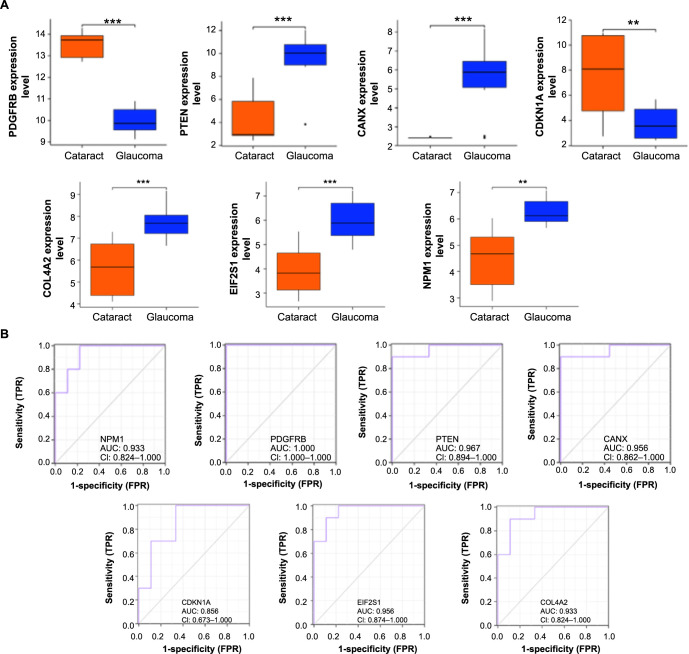
**Expression level validation and ROC curve analysis of seven genes.** (A) Expression analysis of seven overlapping genes in different samples of the GSE101727 dataset. The orange box line represents the cataract group and the blue box line represents the glaucoma group; (B) ROC curve analysis of seven genes in the GSE101727 data sample. **P < 0.01, ***P < 0.001. ROC: Receiver operating characteristic; FPR: False positive rate; TPR: True positive rate.

### Enrichment analysis of seven overlapping genes

Among 313 aging-related genes and 1713 DEGs, we identified 19 overlapping genes (Figure 2A). Next, we constructed a PPI network of 19 genes (Figure 2B) and obtained a network containing seven genes (*PDGFRB, CDKN1A, COL4A2, NPM1, CANX, PTEN, EIF2S1*). The cluster distribution of these seven genes in the 20 samples is shown in Figure 2C. To verify the biological functions of these genes, we performed GO term and KEGG pathway analysis on them, respectively. In the GO enrichment results, the enrichment items of genes were aging (BP, GO: 0007568), regulation of smooth muscle cell proliferation (BP, GO: 0048660), smooth muscle cell proliferation (BP, GO: 0048659), dendritic spine (CC, GO:0043197), neuronal spine (CC, GO: 0044309), Schmidt–Lanterman notch (CC, GO: 0043220), platelet-derived growth factor receptor binding (MF, GO: 0005161), and ionotropic glutamate receptor binding (MF, GO: 0035255) (Figure 2D and Table S2). The enriched pathways of these seven genes in KEGG included melanoma, glioma, human papillomavirus infection, etc. (Figure 2E).

### Analysis of differential expression and clinical value of aging-related genes in cataracts

Expression analysis showed that among the seven overlapping genes, except for *PDGFRB* and *CDKN1A*, which had higher expression levels in the GSE101727 case group, the other five genes had lower expression levels in the case group (Figure 3A). Based on this, it was speculated that *PDGFRB* and *CDKN1A* were promoting genes, and *PTEN, CANX, COL4A2, EIF2S1,* and *NPM1* were suppressing genes in cataract pathogenesis. We next assessed the clinical prognostic value of seven genes for cataract patients according to the ROC curve. The results showed that these genes all had strong diagnostic predictive ability (Figure 3B). Among them, *PDGFRB* had the highest AUC value of 1, followed by *PTEN* (AUC ═ 0.967), *CANX* and *EIF2S1* (AUC ═ 0.956), *NPM1* and *COL4A2* (AUC ═ 0.933), and, finally, *CDKN1A* (AUC ═ 0.856).

**Figure 4.**
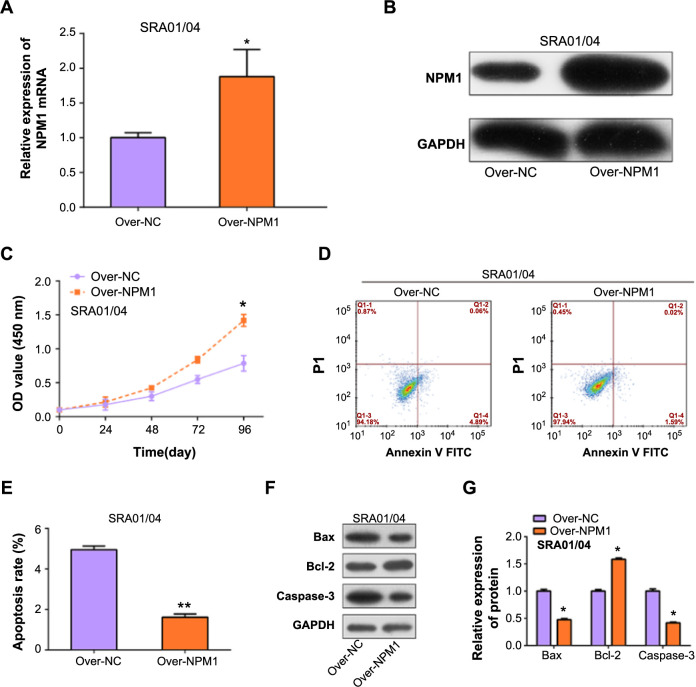
**Impact of *NPM1* overexpression on proliferation and apoptosis in SRA01/04 cells.** (A and B) Evaluation of transfection efficiency of the *NPM1* overexpression plasmid in SRA01/04 cells through (A) qRT-PCR and (B) western blot analyses; (C) CCK-8 assay assessing cell proliferation in SRA01/04 cells with *NPM1* overexpression; (D and E) Flow cytometry analysis of apoptosis in SRA01/04 cells with *NPM1* overexpression; (F and G) Western blot analysis of apoptosis-related proteins (Bax, Bcl-2, and Caspase-3) in SRA01/04 cells with *NPM1* overexpression. *P < 0.05, **P < 0.01. NPM1: Nucleophosmin 1; qRT-PCR: Quantitative real-time PCR; CCK-8: Cell counting kit-8.

**Figure 5.**
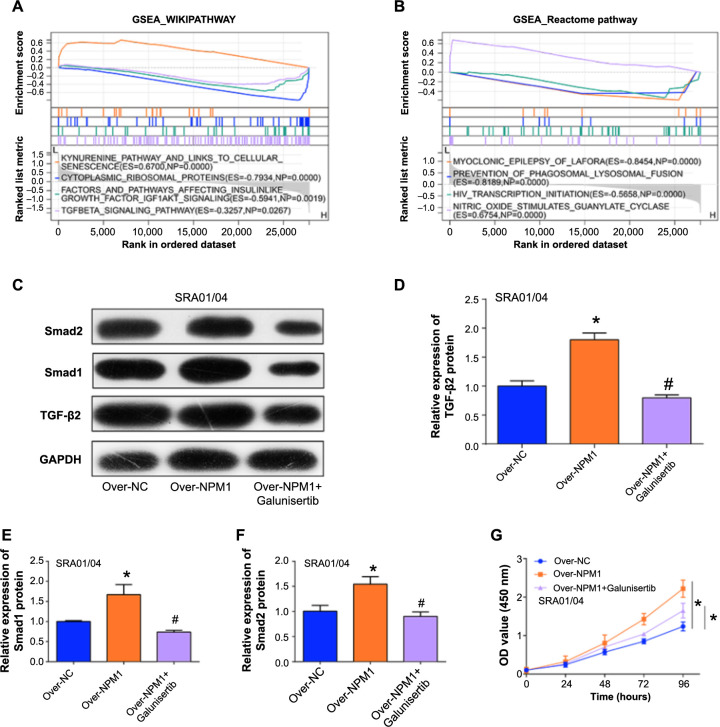
***NPM1* modulates proliferation in SRA01/04 cells through the TGF-β signaling pathway.** (A) Results of GSEA-Wikipathway enrichment analysis of *NPM1*; (B) Results of GSEA-Reactome pathway enrichment analysis of *NPM1*; (C–F) Western blot analysis and corresponding bar graphs of protein expression levels of TGF-β, Smad1, and Smad2 after *NPM1* overexpression along with introduction of TGF-βRI inhibitor; (G) CCK-8 validation of the effects of Over-NC, Over-*NPM1*, and Over-*NPM1* + galunisertib on the proliferation of SRA01/04 cells. *P < 0.05. NPM1: Nucleophosmin 1; TGF-β: Transforming growth factor-β; GSEA: Gene set enrichment analysis; CCK-8: Cell counting kit-8.

### *NPM1* overexpression promotes SRA01/04 cell proliferation and inhibits apoptosis

qRT-PCR and WB analyses detected significant *NPM1* overexpression transfection efficiency in SRA01/04 cells (Figure 4A and 4B). We used CCK-8 assay (Figure 4C) and flow cytometry (Figure 4D and 4E) on SRA01/04 cells to study the impacts of *NPM1* overexpression on cell proliferation and apoptosis. Results unequivocally indicate that *NPM1* overexpression stimulates cell proliferation while concurrently suppressing apoptosis. WB analysis (Figure 4F and 4G) of apoptosis-related proteins revealed a decrease in Bax and Caspase-3, pro-apoptotic proteins, and an elevation in the anti-apoptotic protein Bcl-2. These experimental results demonstrated that *NPM1* overexpression significantly affects the apoptosis as well as the proliferation of lens epithelial cells.

### *NPM1* modulates proliferation in lens epithelial cells via the TGF-β signaling pathway

Through the GSEA database, we conducted Wikipathway and Reactome pathway enrichment analyses on the hub gene of this study, *NPM1*, and obtained some enriched pathways including cytoplasmic ribosomal proteins, TGF-β signaling pathway, and HIV transcription initiation (Figure 5A and 5B). Among them, we selected the TGF-β signaling pathway for subsequent experimental analysis. To further understand the link between *NPM1* and the TGF-β signaling pathway, we conducted analyses on TGF-β2 and Smad1/2, the key proteins associated with this pathway. By utilizing WB analysis and visualized bar graphs (Figure 5C–5F), we observed that *NPM1* overexpression enhances the protein expression of TGF-β2, Smad1, and Smad2. Conversely, the introduction of the corresponding inhibitor, galunisertib, resulted in a decrease in protein expression levels of TGF-β2 and Smad1/2. Subsequently, a CCK-8 assay was employed to analyze the proliferation of SRA01/04 cells with *NPM1* overexpression and the addition of the inhibitor (Figure 5G). The results revealed a downward trend in cell proliferation induced by *NPM1* overexpression after the introduction of the inhibitor. These findings suggested that *NPM1* regulates the proliferation of SRA01/04 cells through the TGF-β signaling pathway.

### TGF-**β**2 induces autophagy as well as EMT in SRA01/04 cells

**Figure 6.**
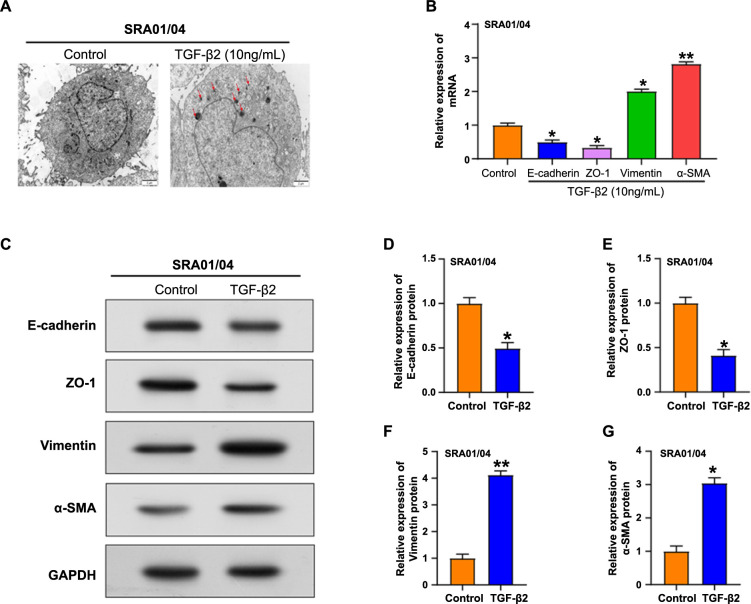
**Overexpression of *NPM1* affects the apoptosis and proliferation of SRA01/04 cells.** (A) Transmission electron microscopy observation of morphological changes in autophagic vesicles of SRA01/04 cells treated with TGF-β2 (10 ng/mL); (B) qRT-PCR analysis of E-cadherin, ZO-1, vimentin, and α-SMA expression in SRA01/04 cells treated with TGF-β2; (C–G) Western blot analysis of EMT-associated proteins (E-cadherin, ZO-1, vimentin, and α-SMA) in SRA01/04 cells treated with TGF-β2. *P < 0.05, **P < 0.01. NPM1: Nucleophosmin 1; TEM: Transmission electron microscopy; TGF-β2: Transforming growth factor-β2; qRT-PCR: Quantitative real-time PCR; EMT: Epithelial–mesenchymal transition.

TEM investigation of SRA01/04 cells after TGF-β2 treatment demonstrated the existence of autophagic vacuoles, a sign of active autophagy (Figure 6A). qRT-PCR was used to identify the expression of epithelial markers E-cadherin and ZO-1, as well as interstitial markers α-SMA and vimentin. Remarkably, compared to control cells, TGF-β2-treated SRA01/04 cells exhibited upregulation of vimentin and α-SMA as well as downregulation of ZO1 and E-cadherin (Figure 6B). In addition, WB analysis confirmed the qRT-PCR results (Figure 6C–6G). Based on changes in cell morphology and expression of EMT-related markers, our results demonstrated the ability of TGF-β2 to induce EMT in SRA01/04 cells.

### *NPM1* knockdown reverses TGF-**β**2-induced EMT in SRA01/04 cells

**Figure 7.**
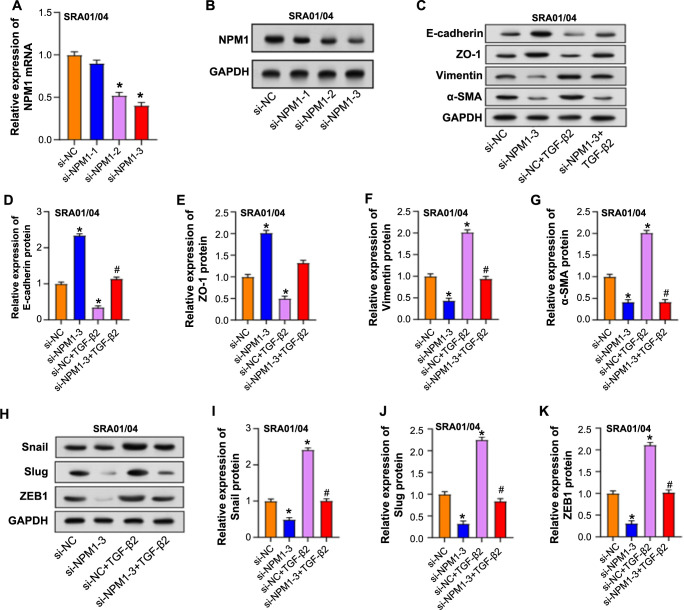
**Reversal of TGF-β2-induced EMT by *NPM1* knockdown in SRA01/04 cells.** (A and B) qRT-PCR and western blot analysis to evaluate the knockdown efficiency of three si-*NPM1*; (C–G) Western blot analysis of protein expression changes of E-cadherin, ZO-1, vimentin, and α-SMA in SRA01/04 cells after si-*NPM1*-3 and TGF-β2 treatment alone or combined; (H–K) Western blot analysis of changes in Snail, Slug, and ZEB1 protein levels in SRA01/04 cells after treatment with si-*NPM1*-3 and TGF-β2 alone or in combination. *P < 0.05 vs si-NC, ^#^*P* < 0.05 vs si-NC+ TGF-β2. TGF-β2: Transforming growth factor-β2; EMT: Epithelial–mesenchymal transition; NPM1: Nucleophosmin 1; qRT-PCR: Quantitative real-time PCR.

The knockdown efficiency of three siRNAs was evaluated by qRT-PCR and WB analysis (Figure 7A and 7B), among which si-*NPM1*-3 exhibited the most efficient knockdown and was selected for subsequent experiments. By WB analysis, we examined the expression changes of EMT-related proteins after the introduction of si-*NPM1*-3 (Figure 7C–7G). Comparing the si-*NPM1*-3 group to the control, there was a decrease in vimentin and α-SMA levels and an increase in ZO-1 and E-cadherin expression. Furthermore, the effects of TGF-β2 treatment were reversed by si-*NPM1*-3, which reduced the expression of vimentin and α-SMA and increased ZO-1 and E-cadherin levels. Afterward, we further analyzed the changes in transcription factors closely related to the EMT process through WB experiments. The protein levels of Snail, Slug, and ZEB1 were downregulated after *NPM1* knockdown. Induction of TGF-β2 alone elevated the levels of these proteins. However, simultaneous application of TGF-β2 and si-*NPM1*-3 reversed the upregulatory effect of TGF-β on the expression of EMT-related transcription factors (Figure 7H–7K). Our results confirmed that *NPM1* silencing can effectively reverse the TGF-β2-induced EMT process in SRA01/04 cells, emphasizing the regulatory function of *NPM1* in cells.

### miR-429 modulates *NPM1* expression and regulates apoptosis in SRA01/04 cells

**Figure 8.**
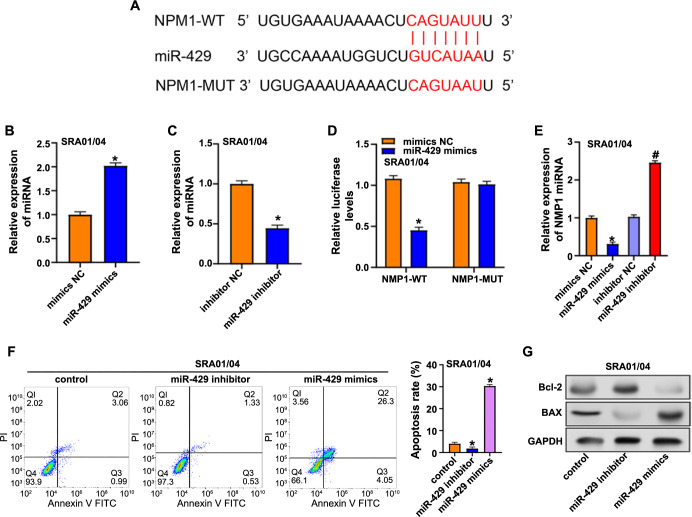
**MiR-429 regulation of *NPM1* and apoptosis in SRA01/04 cells.** (A) ENCORI prediction showing the binding sites of miR-429 on the *NPM1* 3’UTR; (B and C) Transfection efficiencies of miR-429 mimic and inhibitor assessed by qRT-PCR; (D) Dual luciferase assay activity of miR-429 mimics in WT luciferase and MUT luciferase; (E) Flow cytometry to detect the effects of miR-429 mimics and miR-429 inhibitor on cell apoptosis; (G) After transfection with miR-429 mimics and miR-429 inhibitor, changes in apoptosis-related protein levels were analyzed by western blot. *P < 0.05. NPM1: Nucleophosmin 1; qRT-PCR: Quantitative real-time PCR; WT: Wildtype; MUT: Mutated.

To further elucidate the upstream regulatory mechanisms of *NPM1*, we employed ENCORI (https://rnasysu.com/encori/) to predict binding sites for *NPM1* (Figure 8A) and found that the *NPM1* 3’UTR and miR-429 interacted, as indicated by significant parameters, including TDMDscore (0.5055), phylop (4.336), and Pan-Cancer (13) scores. Subsequently, transfection efficiencies of miR-429 mimics and inhibitors were assessed through qRT-PCR (Figure 8B and 8C). Dual-luciferase assay results (Figure 8D) demonstrated that relative to the control group, the miR-429 mimics significantly reduced WT luciferase activity, validating the accuracy of the prediction, while no significant impact was observed on MUT, indicating specificity. *NPM1* and miR-429 were shown to be negatively correlated by qRT-PCR investigation of the effects of miR-429 overexpression and knockdown on *NPM1* (Figure 8E). Exploring the impact of miR-429 on lens epithelial cells apoptosis using flow cytometry revealed a reduction in apoptosis with miR-429 inhibitor and an increase with miR-429 mimics than the control group (Figure 8F). WB analysis of apoptosis-related proteins confirmed these findings. In SRA01/04 cells, the miR-429 mimic decreased Bcl-2 expression while promoting Bax expression, but the miR-429 inhibitor had the reverse effect (Figure 8G). In conjunction with previous experiments, our findings demonstrated that miR-429 targets and inhibits *NPM1* expression, influencing cellular apoptosis in lens epithelial cells.

### miR-429 regulates hydrogen peroxide-induced autophagy in SRA01/04 cells

**Figure 9.**
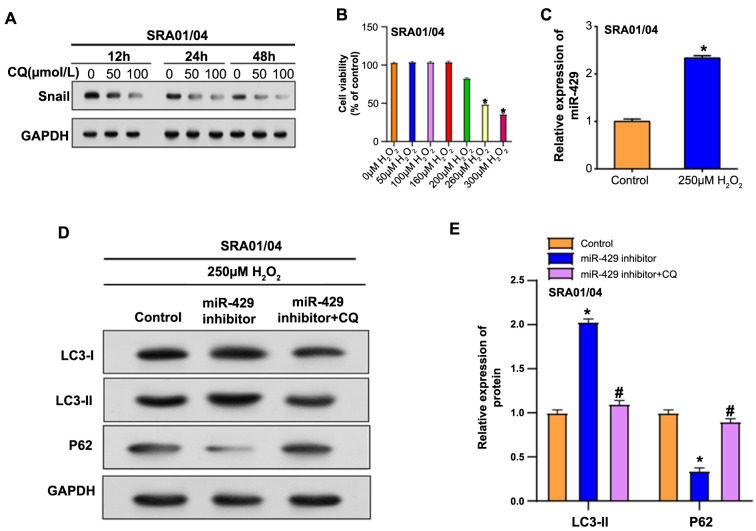
**miR-429 regulates hydrogen peroxide-induced autophagy in SRA01/04 cells under oxidative stress.** (A) Western blot analysis of Snail expression in SRA01/04 cells treated with varying concentrations of CQ for 12, 24, and 48 h; (B) MTT assay assessing the viability of SRA01/04 cells treated with different concentrations of hydrogen peroxide for 24 h; (C) qRT-PCR analysis of miR-429 mRNA expression in SRA01/04 cells after treatment with 250 µmol of hydrogen peroxide; (D) Western blot analysis of the levels of autophagy proteins LC3-I, LC3-II, and P62 in SRA01/04 cells after treatment with 250 µmol of hydrogen peroxide and introduction of miR-429 and CQ; (E) LC3-II and P62 protein levels were visualized using Image J. *P < 0.05 vs si-NC, ^#^*P* < 0.05 vs si-NC + TGF-β2. CQ: Chloroquine; qRT-PCR: Quantitative real-time PCR.

Through the WB assay, we examined the expression levels of Snail in SRA01/04 cells treated with varying concentrations of CQ, a drug influencing autophagy, at 12, 24, and 48 h (Figure 9A). Our findings indicate that CQ suppresses Snail expression in SRA01/04 cells, with a more pronounced inhibitory effect observed at higher concentrations and longer durations. Building upon the relationship between autophagy and Snail in EMT, we investigated whether autophagy influenced cataract development. Cell viability of SRA01/04 cells exposed to different doses of hydrogen peroxide over 24 h was determined by MTT assay (Figure 9B), with 250 µM determined as the optimal concentration for further experimentation. After treating SRA01/04 cells with 250 µM of hydrogen peroxide for 24 h, there was a substantial elevation of miR-429 expression relative to the control group, according to a subsequent qRT-PCR study. Further investigations involved WB analysis of autophagy protein levels (Figure 9D and 9E) in SRA01/04 cells after 24 h of 250-µM hydrogen peroxide treatment. Interestingly, the miR-429 inhibitor upregulated LC3-II expression and downregulated P62 protein levels. Nevertheless, when CQ was added after the miR-429 inhibitor, these changes were reversed to normal levels, indicating blocked autophagic flux.

### Interaction of miR-429/*NPM1* axis and TGF-β2 in regulating EMT and autophagy in SRA01/04 cells

**Figure 10.**
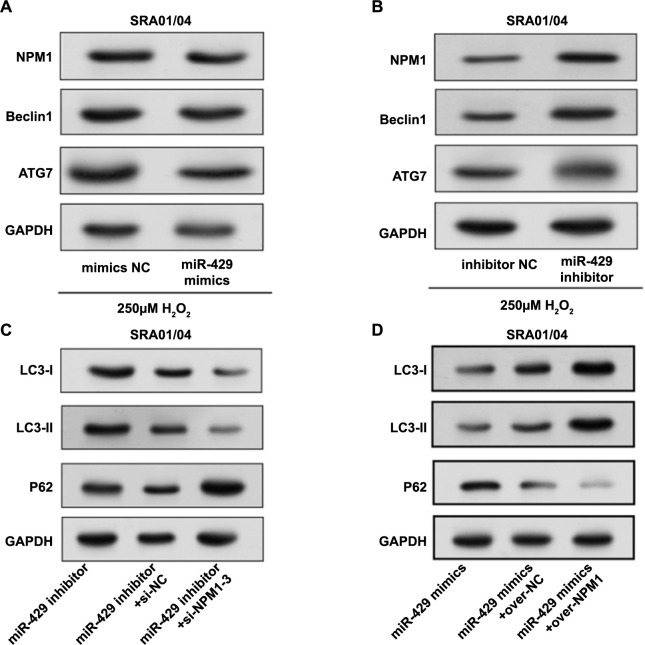
**Regulation of EMT and autophagy in SRA01/04 cells by the miR-492/*NPM1* axis under oxidative stress conditions.** Western blot detected protein levels of NPM1, ATG7, and Beclin1 in SRA01/04 cells after introducing miR-429 (A) mimics and (B) inhibitor under oxidative stress conditions; (C) Western blot analysis of LC3-I, LC3-II, and P62 protein expression levels in SRA01/04 cells after miR-429 and *NPM1* knockdown; (D) Western blot analysis of LC3-I, LC3-II, and P62 protein expression levels in SRA01/04 cells after overexpression treatment of miR-429 and NPM1. EMT: Epithelial–mesenchymal transition; NPM1: Nucleophosmin 1.

**Figure 11.**
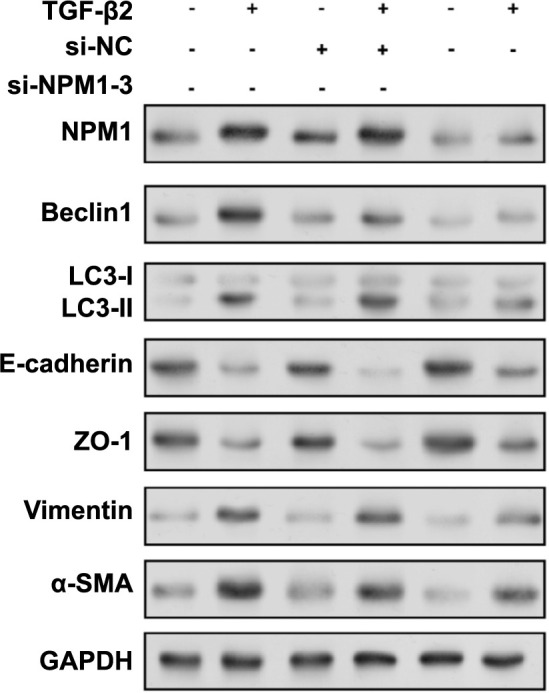
**Effects of TGF-β2 and si-*NPM1*-3 on autophagy and EMT markers in lens epithelial cells.** Western blot analysis of NPM1, autophagy markers (Beclin1, LC3-I, LC3-II) and EMT markers after treating cells with TGF-β2 and/or transfecting cells with si-NPM1-3 or si-NC (negative control). The expression levels of substances (E-cadherin, ZO-1, vimentin, α-SMA). “+” indicates the presence of the specified treatment or transfection, and “-” indicates the absence of the specified treatment or transfection. TGF-β2: Transforming growth factor-β2; NPM1: Nucleophosmin 1; EMT: Epithelial–mesenchymal transition.

In SRA01/04 cells, miR-429 adversely controlled the levels of NPM1 protein, autophagy protein ATG7, and Beclin1 under oxidative stress (Figure 10A and 10B). Subsequently, the regulation of autophagy proteins in SRA01/04 cells by the differential expression of miR-429 and *NPM1* was also analyzed. From the results in Figure 10C and 10D, *NPM1* downregulation restored the autophagy activity promoted by miR-429 inhibitors, while *NPM1* overexpression reversed the autophagy activity inhibited by miR-429 mimics. Therefore, it can be concluded that miR-429 regulates autophagy by inhibiting *NPM1* in SRA01/04 cells. Next, we studied the effect of *NPM1* knockdown on the expression of EMT-related proteins and autophagy proteins in the presence or absence of TGF-β2 (Figure 11). Following TGF-β2 treatment, NPM1, Beclin1, LC3-II, vimentin, and α-SMA expressions were upregulated in SRA01/04 cells, but E-cadherin and ZO-1 levels were downregulated. After *NPM1* knockdown, NPM1, Beclin1, LC3-II, vimentin, and α-SMA were downregulated, while E-cadherin and ZO-1 were upregulated. When TGF-β2 and si-*NPM1*-3 were used in combination, these protein levels were regulated, suggesting an interaction between TGF-β2-induced EMT and *NPM1*-mediated autophagy pathways. Based on the above WB analysis, we concluded that TGF-β2-induced EMT in SRA01/04 cells is regulated by the miR-492/*NPM1* axis, affecting EMT and autophagy processes.

## Discussion

As a common disease in the elderly, cataracts have replaced trachoma as the first blinding disease. Age, metabolic irregularities, genetics, local dietary deficiencies, and systemic metabolic illnesses like diabetes are some of the reasons for cataracts [36, 37]. With the advance of medical technology, cataract treatment methods and drugs have become increasingly diversified. However, the majority of cataract patients still face difficulties in early diagnosis and timely treatment. Therefore, the study on early diagnosis and new therapies has certain clinical significance for cataract patients. During the bioinformatics analysis on samples in the GSE101727 dataset and aging-related genes, we performed enrichment analysis of DEGs. DEGs were found to be enriched in aspects, such as histone deacetylation, ribosome, oxidative phosphorylation, HIF-1 signaling pathway, etc., indicating that DEGs may affect cataract progression by regulating these enrichment items and pathways. Researchers have previously studied its relationship with cataracts. A family of proteases known as histone deacetylases is crucial for the structural modification of chromatin and the control of gene translation [38]. Wang et al. [39] found that UV-B induced *ERCC6* expression in cataractous lens epithelial cells, resulting in epigenetic changes in DNA hypermethylation and histone deacetylation. In cellular oxidation, ATP generation is accompanied by a process known as oxidative phosphorylation, and it is important in the mechanism of cataract formation with the aging of the lens [40]. Ribosomes are organelles capable of translating DNA into proteins, and the study by Turi et al. [41] demonstrated a correlation between impaired ribosome biosynthesis, cancer mechanisms, and aging. These findings emphasize the complexities of cataract pathogenesis, pointing out the intricacies of genetic and metabolic components in its development, as well as the possibility of novel diagnostic and therapeutic treatments targeting molecular pathways.

Through the PPI network and DEGs enrichment analyses, we identified seven candidate genes and selected *NPM1* as the central gene for further study. Many studies have demonstrated the involvement of *NPM1* in many disease types, including leukemia and solid tumors, suggesting that *NPM1* may have a role in the course of disease [12, 42]. Nevertheless, the role of *NPM1* in ocular diseases, particularly cataracts, remains largely uncharted. Our bioinformatics research revealed that *NPM1* is elevated in cataract samples, implying that it plays a role in cataract pathogenesis. Subsequent in vitro cell tests revealed that *NPM1* therapy increased cell viability while decreasing apoptosis. Caspase-3, Bcl-2, and Bax are important enzymes in mitochondria-mediated apoptosis [43]. Studies have shown that the Bcl-2/Bax ratio influences cell survival and regulates the rate of apoptosis [44, 45]. Furthermore, caspase-3 plays a crucial role in apoptosis by removing inhibitors to enable feedback amplification [46]. According to our findings, overexpression of *NPM1* can regulate the production of Bcl-2, Caspase-3, and Bax, which in turn affects the speed human lens epithelial cells undergo apoptosis. Our findings provide new insights into the pathogenesis of cataracts and point to potential paths for intervention, underscoring the importance of *NPM1* in the cellular processes of cataract formation.

We found the TGF-β signaling pathway to be one of the primary pathways for *NPM1* enrichment. This signaling system has been shown to have a function in the creation and maintenance of the eye lens [47, 48]. TGF-β family is critical for regulating cell differentiation and other physiological processes and its dysregulation is linked to developmental defects, illness, cancer, and fibrosis [49, 50]. The TGF beta/Smad signaling system, which regulates cell division and proliferation, depends on the proteins Smad1/2 [51]. Consequently, we investigated the TGF-β2 and Smad1/2 proteins associated with the TGF-β signaling cascade. Additionally, to delve deeper into the relationship between *NPM1* and TGF-β, we carried out tests using a TGF-β inhibitor (galunisertib). Galunisertib is a selective inhibitor of TGF-β receptor I that can disrupt the TGF-β signaling cascade, an essential regulator of cell proliferation and differentiation [52]. Research examining pharmacological methods through TGF-β signaling blockade underscores the potential application of galunisertib in human clinical trials [53]. Our findings demonstrated that the addition of a TGF-βRI inhibitor to overexpressed *NPM1* resulted in downregulated TGF-β2 and Smad1/2 protein expression levels as well as inhibition of cell proliferation.

TGF-β2 is a significant inducer of EMT and is intimately connected to it [54]. Individuals with high-grade myopia and cataract showed higher levels of TGF-β2 in their aqueous humor, indicating that this protein may have a role in the initiation and progression of high-grade myopia [55]. Furthermore, research has revealed that *FOXM1* expression is elevated in the lens tissue of cataract patients, which results in TGF-β2 damaging human lens epithelial cells by boosting VEGFA production and initiating the MAPK signaling pathway [56]. EMT is a phenomenon related to cell morphology, cell structure, and cell migration ability [57]. There are currently many studies on the EMT process. An important study shows that EMT is a key process associated with retinal pathologies, such as diabetic retinopathy, AMD, and proliferative vitreoretinopathy [58]. Dysregulation of EMT in the RPE due to factors such as oxidative stress may disrupt cellular function [59]. Another study showed that in fibrotic eye diseases, the EMT process causes cells to differentiate into myofibroblasts under the influence of TGF-β, leading to the pathological formation of fibrotic tissue [60]. Current research indicates that the activation of the complement system—particularly via the C5a-C5aR pathway, thus inducing EMT in retinal pigment epithelial cells—is a key factor in the progression of subretinal fibrosis linked to neovascular AMD [25]. We found that TGF-β2 may promote EMT in SRA01/04 cells, and *NPM1* knockdown can reverse TGF-β2-induced EMT in lens epithelial cells.

miR-429 is critical for many biological processes, including cell proliferation, differentiation, migration, and embryonic development [61]. It emerges as a key orchestrator in the EMT process, with research indicating its role in inhibiting hepatocellular carcinoma cell migration by targeting RAB23 and reversing EMT [62]. Additionally, autophagy, a cellular process involving the degradation and recycling of cellular components, has been increasingly recognized in the pathogenesis of cataracts [63]. Dysregulated autophagy contributes to lens opacification, a hallmark of cataract formation, by affecting lens epithelial cell homeostasis and response to stressors like oxidative damage [64, 65]. Recent investigations underscored the pivotal function of autophagy in fostering TGF-β2-induced EMT in lens epithelial cells. A novel study in the context of diabetic cataracts revealed that elevated glucose levels induced EMT in lens epithelial cells through the activation of Jagged1/Notch1/NICD/Snail signaling and inhibition of autophagy via the AKT/mTOR/ULK1 pathway [66]. miR-429 has been confirmed to play a significant role in various physiological and pathological processes. Notably, analyses such as TDMDscore (0.5055), phylop (4.336), and Pan-Cancer (13) indicate its stronger RNA-RBP binding with NPM1, higher conservation across different species, and its involvement in diverse diseases and cancers. Therefore, the selection of miR-429 as a research target is not only based on database predictions but also considers its biological importance and existing literature support. This rationale underscores the significance of investigating miR-429 in our study. Our experiments unveiled a reciprocal relationship between miR-429 and *NPM1*. The exploration of associations between miR-429 and autophagy-related proteins clarified the intricate interplay between miR-429 and autophagy within lens epithelial cells. Lastly, we investigated the regulatory role of the miR-429/*NPM1* axis in the EMT and autophagy processes produced by TGF-β2 in lens epithelial cells by evaluating the expression patterns of autophagy-related and EMT-related proteins.

## Conclusion

In conclusion, the miR-429/*NPM1* axis, according to our findings, was critical for modulating autophagy and EMT in lens epithelial cells, especially when oxidative stress is present. We observed that miR-429 regulated *NPM1* expression and had an effect on autophagy and apoptosis. Furthermore, the fact that the miR-429/*NPM1* axis regulated TGF-β2 effects on EMT-related proteins implies that it might be a promising target for cataract treatment. The rise in autophagy markers and EMT indications following TGF-β2 treatment underscored the complexity of cataract etiology, as does the therapeutic value of concentrating on these molecular interactions.

## Supplemental data

**Table S1:** Primer sequences used in qRT-PCR

**Target**	**Sequence 5′–3′ (forward)**	**Sequence 5′–3′ (reverse)**
*NPM1*	ACTCCACCCTTTTGCTTGGTTT	TTTGTCTCCCCACCATTTCC
E-cadherin	GGCTGGACCGAGAGAGTTACC	CACTTTGAGTGTGGCGATCC
*ZO-1*	CGAGTTGCAATGGTTAACGGA	TCAGGATCAGGACGACTTACTGG
Vimentin	GACCAGAGATGGACAGGTGAT	CGTCTTTTGGGGTGTCAGTTG
*α-SMA*	TTCCAGCCTTCCTTTATCG	TTGGCGTACAGGTCCTTC
miR-429	UAAUACUGUCUGGUAAAACCGU	UUCUCCGAACGUGUCACGUTT
*GAPDH*	AGGTCGGTGTGAACGGATTTG	GGGGTCGTTGATGGCAACA
*U6*	CTCGCTTCGGCAGCACA	AACGCTTCACGAATTTGCGT

**Table S2:** GO enrichment analysis list of six genes

**ONTOLOGY**	**ID**	**Description**	**GeneRatio**	***P* value**	**GeneID**
BP	GO:0007568	aging	6/6	2.46662E-11	CANX/EIF2S1/CDKN1A/PTEN/PDGFRB/COL4A2
BP	GO:0048660	regulation of smooth muscle cell proliferation	3/6	1.42845E-05	CDKN1A/PTEN/PDGFRB
BP	GO:0048659	smooth muscle cell proliferation	3/6	1.47972E-05	CDKN1A/PTEN/PDGFRB
CC	GO:0043197	dendritic spine	2/6	0.001071025	CANX/PTEN
CC	GO:0044309	neuron spine	2/6	0.001096304	CANX/PTEN
CC	GO:0043220	Schmidt-Lanterman incisure	1/6	0.003343123	PTEN
MF	GO:0005161	platelet-derived growth factor receptor binding	2/6	1.00389E-05	PTEN/PDGFRB
MF	GO:0035255	ionotropic glutamate receptor binding	2/6	4.73003E-05	CANX/PTEN

## Data Availability

The datasets used and/or analyzed during the current study are available from the corresponding author on a reasonable request.
